# Transcriptional regulation of *MdmiR285N* microRNA in apple (*Malus x domestica*) and the heterologous plant system *Arabidopsis thaliana*

**DOI:** 10.1038/s41438-020-0321-5

**Published:** 2020-07-01

**Authors:** Valerio Pompili, Stefano Piazza, Mingai Li, Claudio Varotto, Mickael Malnoy

**Affiliations:** 1 Department of Genomics and Biology of Fruit Crops, Research and Innovation Centre, Fondazione Edmund Mach, Via E. Mach 1, San Michele all’Adige, 38010 Italy; 2 Department of Agricultural, Food, Environmental and Animal Sciences, Università degli Studi di Udine, Via delle Scienze 206, Udine, 33100 Italy; 3 Department of Biodiversity and Molecular Ecology, Research and Innovation Centre, Fondazione Edmund Mach, Via E. Mach 1, San Michele all’Adige, 38010 Italy

**Keywords:** Plant molecular biology, Plant signalling

## Abstract

*Malus x domestica* microRNA MdmiR285N is a potential key regulator of plant immunity, as it has been predicted to target 35 RNA transcripts coding for different disease resistance proteins involved in plant defense to pathogens. In this study, the promoter region of *MdmiR285N* was isolated from the apple genome and analyzed in silico to detect potential regulatory regions controlling its transcription. A complex network of putative regulatory elements involved in plant growth and development, and in response to different hormones and stress conditions, was identified. Activity of the *β-Glucoronidase* (*GUS*) reporter gene driven by the promoter of *MdmiR285N* was examined in transgenic apple, demonstrating that *MdmiR285N* was expressed during the vegetative growth phase. Similarly, in transgenic *Arabidopsis thaliana*, spatial and temporal patterns of *GUS* expression revealed that *MdmiR285N* was differentially regulated during seed germination, vegetative phase change, and reproductive development. To elucidate the role of *MdmiR285N* in plant immunity, *MdmiR285N* expression in wild-type apple plants and *GUS* activity in transgenic apple and *Arabidopsis thaliana* plants were monitored in response to *Erwinia amylovora* and *Pseudomonas syringae* pv. *Tomato* DC3000. A significant decrease of MdmiR285N levels and *GUS* expression was observed during host-pathogen infections. Overall, these data suggest that MdmiR285N is involved in the biotic stress response, plant growth, and reproductive development.

## Introduction

Plant microRNAs (miRNAs) are a large subclass of endogenous non-coding RNAs with 20–22 nucleotides taking part in posttranscriptional gene silencing^1,2^. The biogenesis of plant miRNAs occurs in the cell nucleus and involves transcription of *MIRNA* genes, processing of primary miRNA transcripts by DICER-LIKE proteins into miRNA:miRNA* duplexes, and loading of mature miRNA strands into ARGONAUTE-containing RNA-induced silencing complexes (RISC)^1,2^. After RISC loading, the mature miRNA guides the RISC machinery to complementary target sequences on messenger RNAs (mRNA) leading to miRNA-mediated RNA degradation or translational repression^1,2^.

By functioning in RNA silencing and posttranscriptional regulation of gene expression, plant miRNAs coordinate a wide range of biological processes in different cells, tissues, and organs. Since their initial discovery, several functional analyses elucidated the importance of these bio-regulators in almost all aspects of plant growth and development^3,4^, in the crosstalk between phytohormone signaling pathways^5^, and in response to environmental stimuli^6^, abiotic stresses^7^, and pathogen invasions^8^. Besides their relevance in fundamental research, miRNAs are also very important from an applicative point of view to manipulate specific agricultural traits by modulation of plant gene expression^9–11^. Over the last decades, miRNA-mediated crop improvement was successfully achieved by the use of different molecular strategies, including constitutive, stress-induced, or tissue-specific expression of miRNAs^12^, RNA interference^13^, and artificial miRNAs^14^. However, most of these studies have focused on the analysis of miRNAs especially in non-woody plant species, such as *A. thaliana*, rice, wheat, and tomato, while limited investigations have been performed on miRNAs in agronomically and economically important woody plant species.

Apple (*M. x domestica*) is one of the most widely cultivated woody plant species in the world, with a total worldwide production of 85 million tonnes and a global value of 45 billion dollars in 2017^15^. As a result, the scientific attention on this fruit crop has drastically grown in the last years, focusing not only on different aspects of apple horticulture, but also on its fundamental biology, such as the study of miRNAs. To date, ~300 apple miRNAs were deposited in miRBase (www.mirbase.org, release 22.1: October 2018). Some studies were performed to identify apple miRNAs involved in the regulation of plant tissue development^16–18^, shoot growth^19,20^, flower induction^20,21^, and fruit production^22,23^. Others focused on the identification of miRNAs associated with apple response to different diseases, such as apple ring rot^24^, *Alternaria* leaf spot^25,26^, *Glomerella* leaf spot^27^, *Valsa* canker^28^, and fire blight^29^. One study reported a series of miRNAs involved in the response of the plant to drought stress^30^. Although a considerable amount of data is now available, many gaps still exist for apple miRNAs research. Indeed, most of the above studies exploited microarray and next-generation sequencing to screen for putatively novel or stress-responsive miRNAs, but very few studies have been published on the functional characterization of the plethora of candidate miRNAs identified. More efforts are thus required to better characterize miRNAs and their functions in this important plant species.

In this study, as part of a long-term goal to identify promising miRNAs for potential genetic improvement of apple, we focused our attention on MdmiR285N, a novel apple miRNA which is 21 nucleotides in length and predicted to target 35 RNA transcripts^29^. The mRNAs putatively regulated by this miRNA code for different disease resistance proteins belonging to the families of Toll-interleukin-1 receptor/nucleotide-binding site/leucine-rich repeat (TIR-NBS-LRR), SUPPRESSOR of NPR-1 CONSTITUTIVE (SNC1), and calcium-dependent protein kinase (CDPK). These resistance proteins are well-known to play key roles in plant response to pathogen infections^31–33^. Within this framework, MdmiR285N was thus hypothesized to act as a crucial regulator of plant immunity. Here, a first characterization of the *MdmiR285N* promoter region was carried out in silico to identify putative *cis*-acting regulatory elements and their cognate transcription factors. After isolation from the *M. x domestica* genome, a 2-kbp promoter region of *MdmiR285N* was analyzed in vivo both in *M. domestica* and *A. thaliana* to examine putative roles of *MdmiR285N* in plant growth, development and especially pathogen resistance. In particular, tissue- and organ-specific expression patterns of *β-glucoronidase* (*GUS*) driven by the promoter of *MdmiR285N* were analyzed in transgenic apple and *A. thaliana* plants. With the aim of elucidating the function of *MdmiR285N* in plant immunity, *MdmiR285N* expression in wild-type apple plants and GUS activity in transgenic apple and *A. thaliana* plants were investigated in response to *Erwinia amylovora* (*E. amylovora*) and *Pseudomonas syringae* (*P. syringae*) pv. *Tomato* DC3000 infections.

## Results

### Selection of transgenic apple and *Arabidopsis thaliana* plants

In this study, *Prom_MdmiR285N::GFP-GUS* transgenic apple (*M. x domestica* cultivar ‘Gala’) and *A. thaliana* (ecotype Columbia-0) lines (PMd and PAt, respectively) were generated by *Agrobacterium tumefaciens* (*A. tumefaciens*)*-*mediated transformations.

For apple, the summary of transformation results is shown in Supplementary Table S1. By infecting 770 leaf explants, five plants were regenerated ~5 months after transformation and cultured on selective medium, thus screened by PCR for T-DNA integration. Four transgenic apple lines were obtained as demonstrated by PCR amplification of *NptII* (the selectable marker of the T-DNA cassette) and lack of *VirG* amplification (therefore free from *A. tumefaciens* contamination), resulting in a transformation efficiency of 0.5%. Among the obtained lines, PMd1 and PMd2 were selected for further analyses. The other two lines showed a severe vitrified phenotype compared with wild-type plants, most likely due to the transformation event (data not shown) and were discarded from further analyses. The two selected lines were characterized for the number of T-DNA integration events by quantifying the copy number (CN) of the *NptII* marker gene. The line PMd1 showed a *NptII* CN mean of 2.01 ± 0.12 which corresponded to two T-DNA integration events (Supplementary Table S1). The line PMd2 showed a *NptII* CN mean of 1.00 ± 0.45, which reflected the presence of a remarkable T-DNA chimeric profile. In fact, for this line the presence of T-DNA chimeric tissues was attributed to a *NptII* CN value lower than 1 in some biological replicates tested.

For *A. thaliana*, the summary of transformation results is shown in Supplementary Table S2. Eight lines showed a single T-DNA insertion event as characterized by a germination ratio Kanamycin^Resistant^:Kanamycin^Susceptible^ significantly not different from 3:1 (*X*^2^ < 3.84, *P* > 0.05, Supplementary Table S2). Among those lines, T4 homozygous seeds of two representative lines (PAt6 and PAt28) were used for further experiments.

### In silico prediction of putative transcription factor binding sites in the promoter of *MdmiR285N* gene

**Fig. 1:**
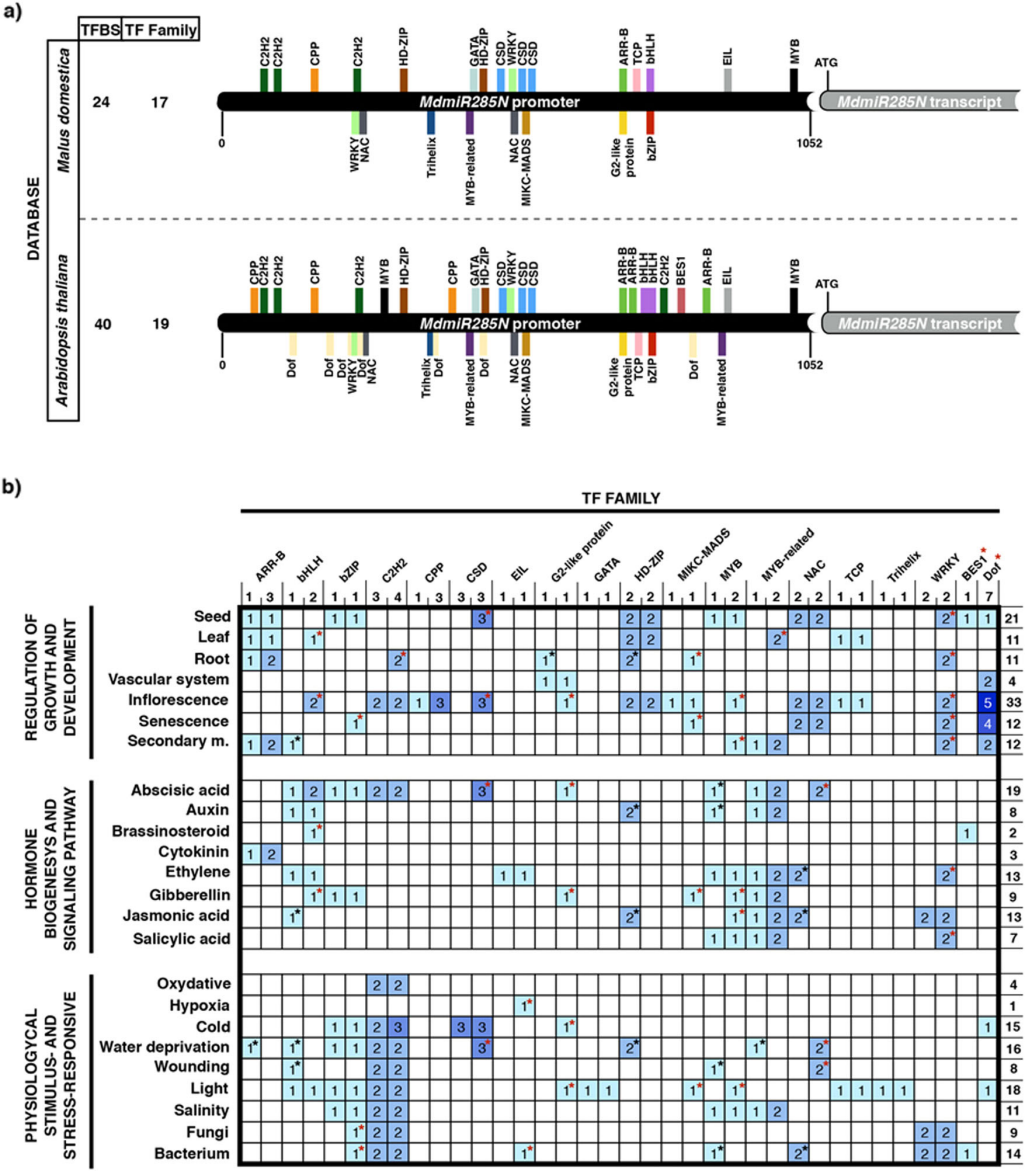
Putative TFBS-based regulatory and functional profile of *MdmiR285N* gene. **a** Summary of TFBSs and corresponding TFs families identified in the promoter region of *MdmiR285N* by PlantPAN 2.0 (http://PlantPAN2.itps.ncku.edu.tw), using *M. x domestica* and *A. thaliana* databases. The spatial distribution of TFBSs along ~1 kb of genomic DNA sequence upstream of the transcription start site (ATG) is reported. Each TFBS is highlighted with a different color based on the corresponding TFs family. **b** Heat map showing the putative TFBS-based functional profile of *MdmiR285N* gene. According to the identified TFBSs and related TFs, TFs families and corresponding biological processes annotated (retrieved by comparing information of both PlantPAN 2.0 and PlantTFDB 5.0 (planttfdb.cbi.pku.edu.cn) databases) are reported, respectively, on the upper and left sides of the heat map. Below each TFs family, the total number of TFBS detected in *M. x domestica* (left column) and *A. thaliana* (right column) is reported. BES1 and Dof families were identified only in *A. thaliana*. For each TFs family, the number of TFBSs recognized by at least one TF associated with a certain biological process is reported within boxes. Black and red asterisks indicate data obtained exclusively in *M. x domestica* or *A. thaliana*, respectively. For each biological process, the total number of associated TFBSs is reported on the right side of the heat map

To identify the likely transcription factor (TF) binding sites (TFBSs) and corresponding TFs of *MdmiR285N* gene, its promoter region was analyzed by the Plant Promoter Analysis Navigator PlantPAN 2.0^34^. When using as reference the database of *M. x domestica* species, 24 unique TFBSs distributed fairly evenly along the *MdmiR285N* promoter sequence were identified (Fig. 1a; Supplementary Table S3). Overall, the TFBSs fell into 17 different TFs families. Interestingly, among them C2H2, CSD, HD-ZIP, NAC, and WRKY families, which are known to play key roles especially in plant development and stress responsiveness^35–39^, were the most frequent, being characterized by multiple TFBS sequences (Fig. 1a). Moreover, consistent results were found when the presence of putative TFBSs was investigated in the heterologous database of *A. thaliana* (Fig. 1 and Supplementary Table S3). However, being *A. thaliana* a model plant species for which the availability of information is significantly greater compared with other plants, the number of putative TFBSs identified (*n* = 40) was higher than that reported in *M. x domestica* species (Fig. 1a). Nevertheless, almost all TFBSs clustered into the same TFs families previously predicted for *M. x domestica*. Indeed, only two TFs families namely BES1 and Dof, which are involved in several plant physiological processes and stress responses^40,41^, were identified only with *A. thaliana* matrixes (Fig. 1a).

Using the available gene ontology information concerning the biological processes associated with each TF detected (Supplementary Table S3), a putative functional profile of *MdmiR285N* gene promoter was generated (Fig. 1b). Results were consistent using either *M. x domestica* or *A. thaliana* matrixes and only few discrepancies, mostly due to the previously mentioned lack of information in apple, were identified. In both cases, *MdmiR285N* promoter was found to be potentially regulated during several biological processes linked to plant growth and development, especially seed formation, vegetative (leaf and root) and reproductive (inflorescence) development, organs senescence and secondary metabolism (Fig. 1b). In addition, a putative functional profile was associated with phytohormones biogenesis and signaling pathways, particularly to those of abscisic acid, ethylene and jasmonic acid (Fig. 1b). Finally, potential responses to multiple physiological stimulus and stress conditions, especially light intensity, water availability, temperature conditions, and bacterial infections, were also predicted (Fig. 1b). Although interesting, information obtained from this in silico analysis should be validated by experimental evidences.

### Tissue- and organ-specific expression pattern of *MdmiR285N* gene in apple and *Arabidopsis thaliana* plants

**Fig. 2:**
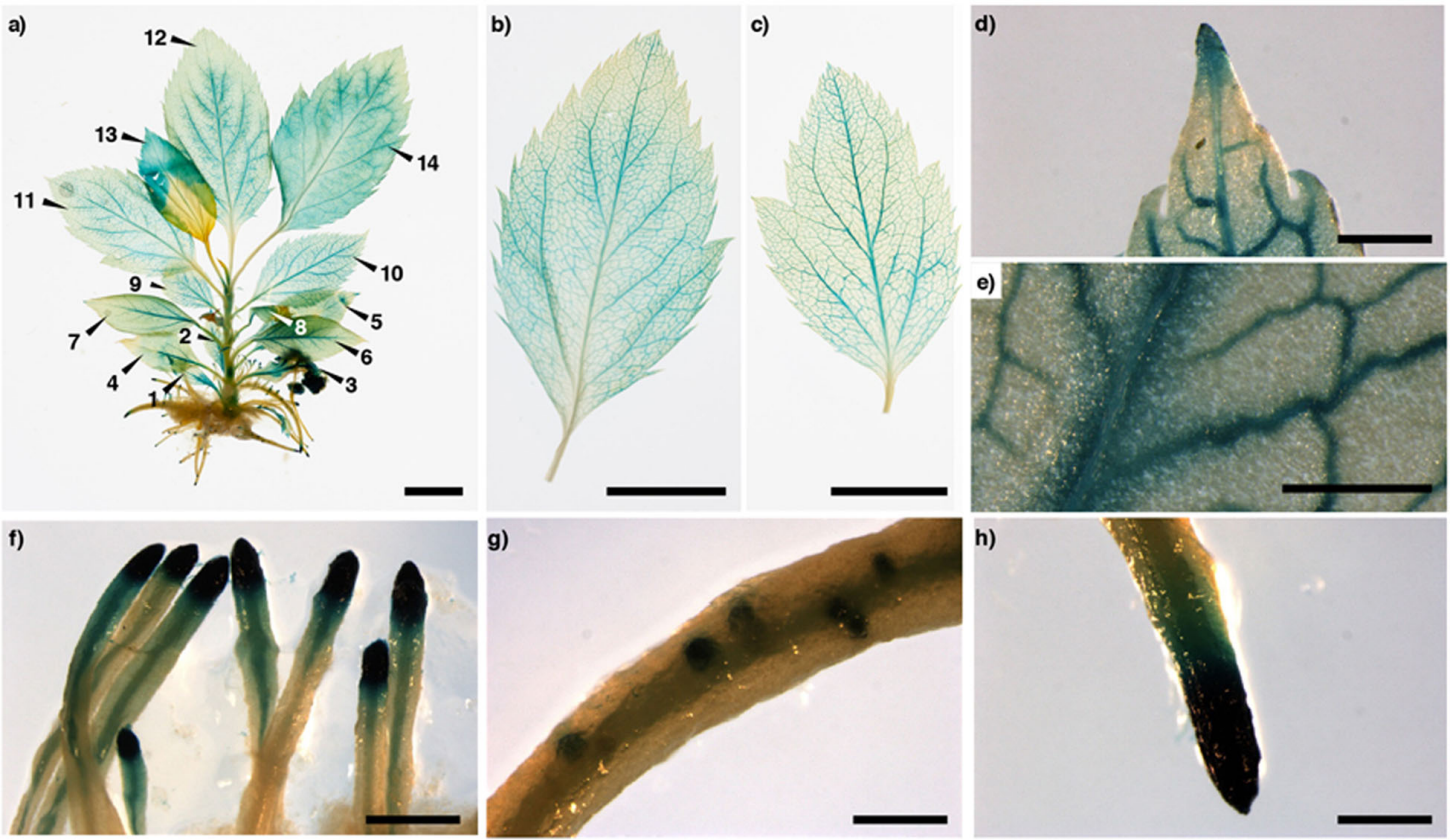
Promoter activity of *MdmiR285N* during vegetative growth in apple. Pictures show the histochemical GUS staining in different tissues and organs of 3-week-old apple plants carrying the construct *Prom_MdmiR285N::GFP-GUS*. **a** Developed apple plant. Numbers indicate the order of leaf appearance. **b**, **c** Young and adult leaf, respectively. **d**, **e** Vascular system in the apical and medial region of the leaf, respectively. **f** Secondary roots. **g** Root buds. **h** Primary root tip. Results (**a**–**h**) were obtained by observations conducted after three independent experiments and are representative of both PMd1 and PMd2 transgenic lines used. In each experiment, five biological replicates/plant line were investigated. Wild-type plants were used as negative control and were never stained following the histochemical GUS protocol (data not shown). Black unit bars indicate 1 cm in (**a**–**c**), 1 mm in (**d**–**h**)

**Fig. 3:**
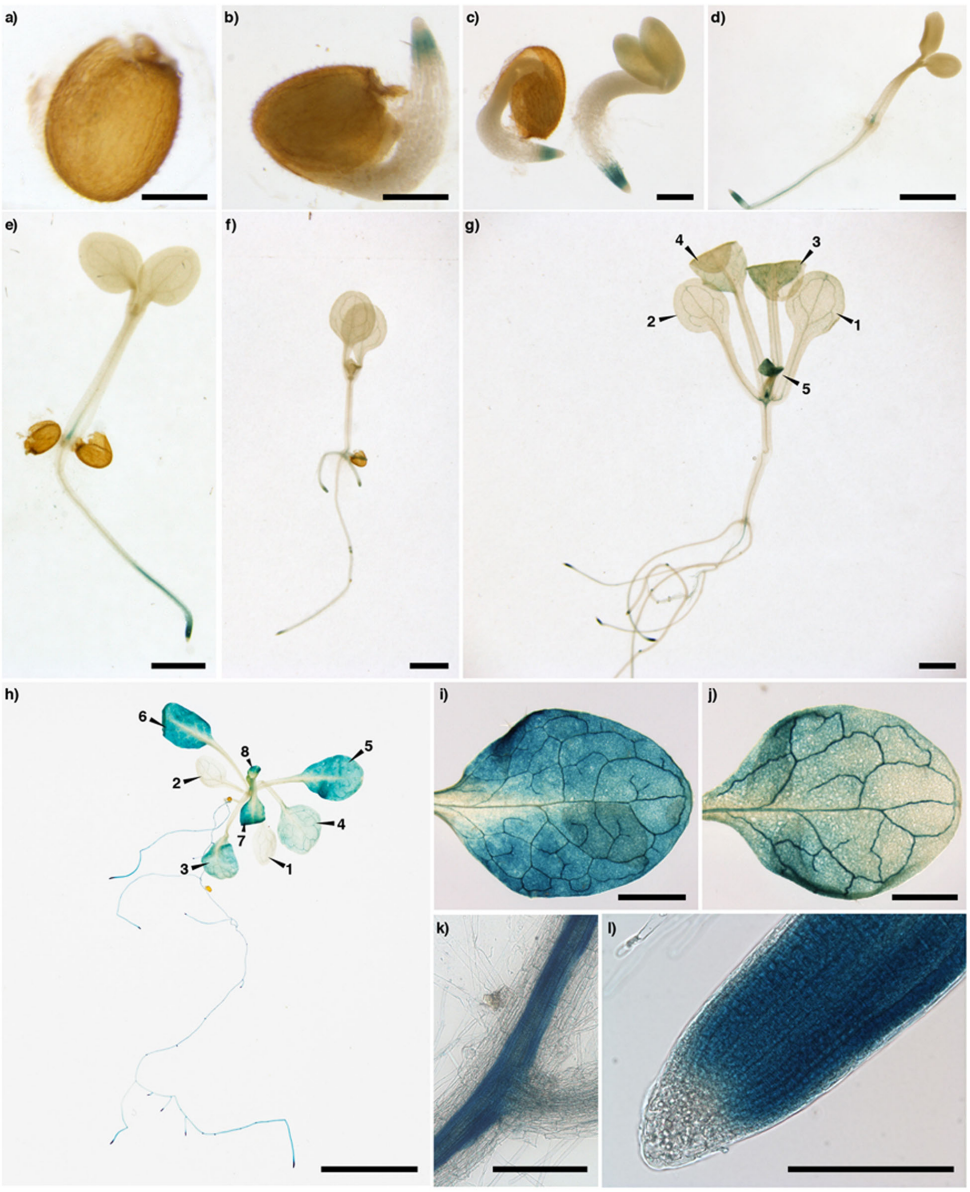
Promoter activity of *MdmiR285N* during seed germination and vegetative development in *Arabidopsis thaliana*. Pictures show the histochemical GUS staining of *A. thaliana* plants carrying the construct *Prom_MdmiR285N::GFP-GUS*. **a** Imbibed seed (1 dap). **b** Emerging radicle from seed coat (2 dap). **c** Emerging hypocotyl and cotyledons from seed coat (3 dap). **d** Germinated seedling (4 dap). **e** Seedling in elongation (5 dap). **f–h** Seedling at the juvenile, intermediate and late phase of vegetative development, respectively (7, 14, 21 dap). In (**g**) and (**h**), numbers indicate the order of leaf appearance. **i–l** Different tissues of *A. thaliana* seedling at the late phase of vegetative development (**h**): young leaf (**i**), adult leaf (**j**), root vascular system (**k**), root tip (**l**). Results (**a**–**l**), representative of both transgenic lines used (PAt6, PAt28), were obtained by observations conducted after three independent experiments. In each experiment, 10 biological replicates/plant line/time point were investigated. Wild-type plants used as negative control were never stained following the histochemical GUS protocol (data not shown). Black unit bars indicate 200 μm in (**a**–**c**), (**k**), (**l**), 1 mm in (**d**–**g**), (**i**), (**j**), and 1 cm in (**h**)

**Fig. 4:**
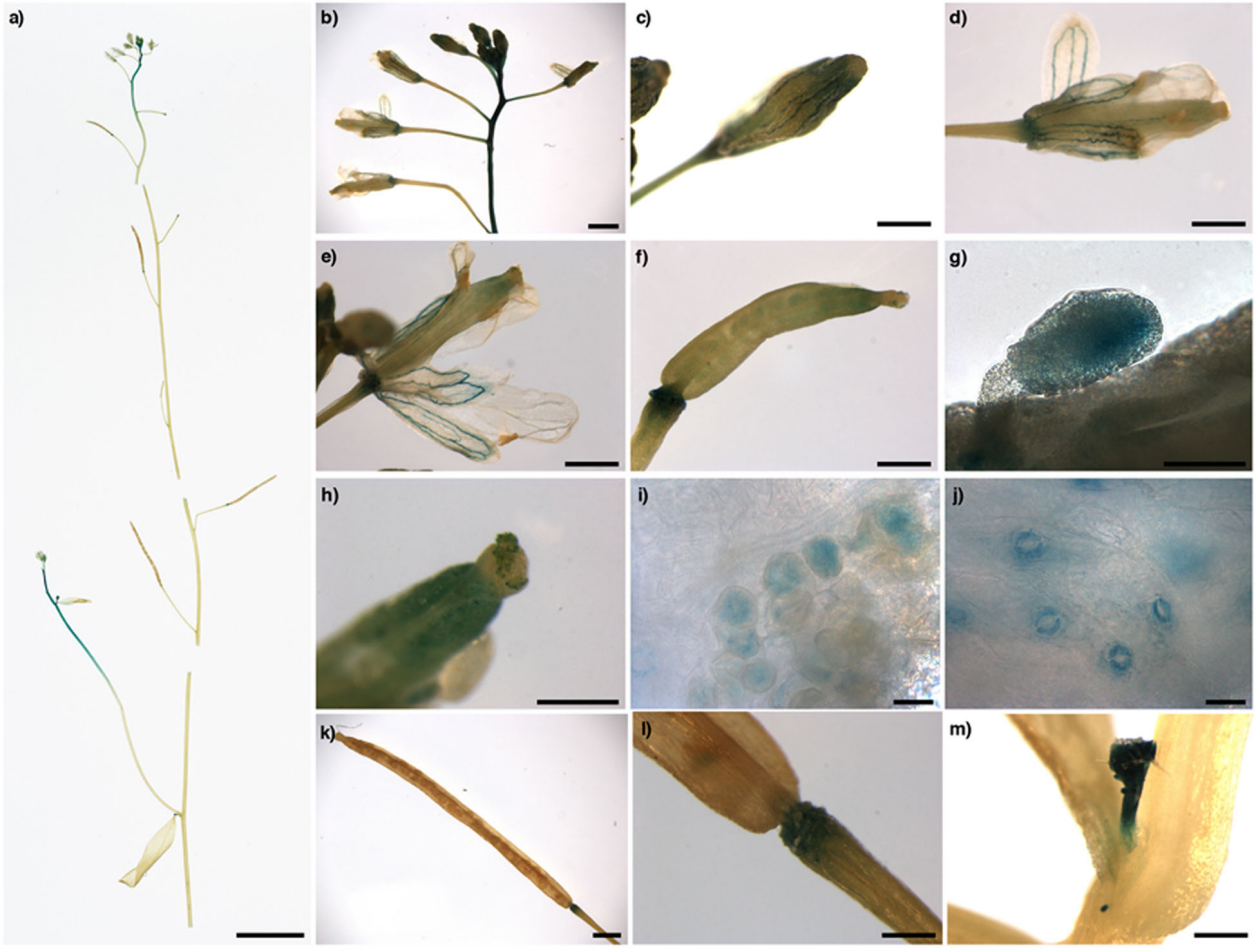
Promoter activity of *MdmiR285N* during reproductive development in *Arabidopsis thaliana*. Pictures show the histochemical GUS staining in reproductive organs of 5-week-old *A. thaliana* plants carrying the construct *Prom_MdmiR285N::GFP-GUS*. **a** Primary inflorescence. **b** Flower set. **c** Closed floral bud. **d**, **e** Mature flowers. **f** Elongating stigma after pollination. **g** Ovule. **h** Stigma apex. **i** Pollen grains on stigma apex. **j** Guard cells of stigma. **k** Mature silique. **l** Abscission zone of mature silique. **m** Axillary bud. Results (**a**–**m**), representative of both AtP6 and AtP28 transgenic lines, were obtained by observations conducted after two independent experiments. In each experiment, the primary inflorescence of five biological replicates/plant line was analyzed. Wild-type plants used as negative control were never stained following the histochemical GUS protocol (data not shown). Black unit bars indicate 1 cm in (**a**), 1 mm in (**b**–**f**), (**k**), 200 μm in (**h**), (**l**), (**m**), and 20 μm in (**g**), (**i**), (**j**)

According to the in silico analysis, to test the hypothesis that *MdmiR285N* is associated with plant growth and development (Fig. 1b), its tissue- and organ-specific expression pattern was investigated in transgenic apple and *A. thaliana* plants by histochemical GUS assay. Since apple was maintained in vitro by clonal propagation, the tissue- and organ-specific expression pattern of *MdmiR285N* was analyzed only during the vegetative growth phase (Fig. 2). However, by using *A. thaliana* as heterologous plant system the activity of *MdmiR285N* promoter was investigated during the entire plant life cycle, including seed germination, juvenile-to-adult vegetative phase change, and reproductive development (Figs. 3, 4).

#### Histochemical expression pattern of *MdmiR285N* during vegetative development in apple

In apple, the histochemical GUS staining revealed that *MdmiR285N* is expressed during the plant vegetative development (Fig. 2). In shoots, GUS expression was detected in the shoot apical meristem (SAM) and in the stem (Fig. 2a). Moreover, all leaves were stained (Fig. 2a), although a stronger GUS signal was observed in young leaves (Fig. 2a, *n* = 9–14; Fig. 2b) compared with adult leaves (Fig. 2a, *n* = 1–8; Fig. 3c). Indeed, while in adult leaves GUS staining was observed only in vascular tissue (Fig. 2c), in young leaves also parenchyma cells proximal to vessels appeared stained (Fig. 2b). In general, the vascular tissue was always stained with no intensity variation between different parts of a leaf (Fig. 2d, e). In roots, strong GUS expression was detected in the tip of primary and secondary roots and in the meristems of emerging lateral roots, in the root vascular system, and in the root elongation zone up to the root maturation region (Fig. 2f–h). Despite the lines PMd1 and PMd2 showed different T-DNA (or *NptII*) copy numbers values (Supplementary Table S1), no significant discrepancy was found in the pattern of GUS activity (data not shown).

Overall, these results were consistent with the previous in silico analysis, according to which the *MdmiR285N* gene promoter appeared to be potentially regulated by a complex network of TFs involved in plant growth and development. Indeed, several of the identified TFBSs, namely those belonging to the ARR-B, G2-like protein, HD-ZIP, and TCP TFs families, were associated with TFs linked to the formation of leaf and root meristems, the morphogenesis of shoot organs, and the development of the vascular system through the regulation of xylem and phloem differentiation (Supplementary Table S3).

#### Histochemical expression pattern of *MdmiR285N* during seed germination and vegetative development in *Arabidopsis thaliana*

In *A. thaliana*, the histochemical GUS assay showed that *MdmiR285N* expression was specifically and differentially regulated during different stages of seed germination and vegetative development.

No *MdmiR285N* promoter-driven GUS expression was observed in imbibed seeds (1 dap: day after plating, Fig. 3a). In the later stages of seed germination, GUS staining was evident in the root apical meristem (RAM) of emerging seedlings (2 dap, Fig. 3b; 3 dap, Fig. 3c). Similarly, the RAM appeared strongly stained in fully germinated and elongating seedlings (4 dap, Fig. 3d; 5 dap, Fig. 3e), although GUS signal was also detected in the root elongation and maturation zones and partially in the root vascular system. The same expression pattern was maintained in the primary and secondary roots of young seedlings (7 dap, Fig. 3f). Besides roots, the aerial part was never stained at any of the developmental stages mentioned above (1–7 dap).

A significant correlation was found between the observed results and the in silico-predicted gene regulatory and functional profile of *MdmiR285N*. Indeed, among the TFs putatively involved in the regulation of *MdmiR285N* promoter, multiple members of the ARR-B, C2H2, MIKC-MADS, and WRKY TFs families were associated with biological processes linked to the regulation of root growth and development (Supplementary Table S3). On the contrary, no functionality of *MdmiR285N* was associated with seed activation and cotyledons development (Supplementary Table S3).

In the later phases of vegetative growth (14 dap, Fig. 3g; 21 dap, Fig. 3h), in multiple organs of the seedling a gradual increase of GUS signal was observed. Its maximum intensity was reached at the late stage of vegetative development (Fig. 3h). Roots (Fig. 3g, h, k, l) were strongly stained according to the pattern previously described, however strong GUS staining was also visible in the SAM (Fig. 3g, h), in the parenchyma cells of leaves (Fig. 3g–j), and in the leaf vascular tissue (Fig. 3g–j). As for apple, at each stage (14 and 21 dap) the intensity of GUS signal was reduced in adult leaves (Fig. 3g, *n* = 1, 2; Fig. 3h, *n* = 1–4; Fig. 3j) compared with young leaves (Fig. 3g, *n* = 3–5; Fig. 3h, *n* = 5–8; Fig. 3i).

Overall in *A. thaliana*, especially in the late phase of vegetative growth (21 dap, Fig. 3h), the tissue- and organ-specific expression pattern of *MdmiR285N* was consistent with that reported in apple (Fig. 2a). This result was not unexpected, as a significant similarity in the regulatory and functional profile of *MdmiR285N* promoter using TFBSs specific for the two plant species was previously observed in silico (Fig. 1). As for apple, also in *A. thaliana* multiple TFs belonging to the ARR-B, G2-like protein, and TCP TFs families were involved in the morphogenesis of shoot and root organs, and the histogenesis of the vascular system (Supplementary Table S3). However, in *A. thaliana* the same biological functions were also observed for members of the C2H2, Dof, MYB-related, and WRKY TFs families (Supplementary Table S3). Interestingly, some TFs of the CSD and MIKC-MADS families were associated with the vegetative to reproductive phase transition of meristems (Supplementary Table S3). This data supported the tremendous increase of *MdmiR285N* expression during the later phases of vegetative growth in *A. thaliana* (Fig. 3g, h).

#### Histochemical expression pattern of *MdmiR285N* during reproductive development in *Arabidopsis thaliana*

In *A. thaliana*, *MdmiR285N* expression was regulated also during the reproductive development (Fig. 4). In the upper part of the primary inflorescence, strong GUS expression was observed in the stalk and flowers (Fig. 4a, b). A close-up examination of close floral buds and fully open flowers revealed that GUS expression was particularly evident in the organ abscission zone, and the veins of flower petals and sepals (Fig. 4c–e). The *MdmiR285N* promoter was also active during the initial developmental stage of the silique. Indeed, GUS signal was observed in the elongating stigma (Fig. 4f), particularly in the abscission zone (Fig. 4f), the ovule (Fig. 4g), the stigma apex (Fig. 4h), the pollen grains on stigma (Fig. 4i), and the guard cells of stigma cover (Fig. 4j). However, as the silique became mature, the promoter activity of *MdmiR285N* was drastically reduced to a level below visual detection, remaining evident only in the abscission zone (Fig. 4k, l). Finally, strong GUS signal was also observed in axillary buds (Fig. 4m).

The obtained results were clearly supported by the previous in silico analysis. Indeed, in *A. thaliana*, except for ARR-B, EIL, GATA, MYB-related, and Trihelix, all the identified TFs families were characterized by TFs associated with the morphogenesis of the inflorescence, the maturation of pollen, the formation of plant ovule, and the development of seeds (Supplementary Table S3). Many other TFs, by acting as regulators of cell aging, were also correlated to the regulation of leaf senescence and the floral organs abscission (Supplementary Table S3).

### Expression profile of *MdmiR285N* gene after host-pathogen infection in apple and *Arabidopsis thaliana* plants

Besides histological experiments, the investigation of putative changes in the expression profile of *MdmiR285N* in response to environmental stimuli, such as bacterial infections, may provide insights into the biological roles of this novel apple miRNA. Thus, the expression pattern of *MdmiR285N* gene was examined in apple and *A. thaliana* plants following inoculation with *E. amylovora* strain Ea273 and *Pst* DC3000, respectively.

**Fig. 5:**
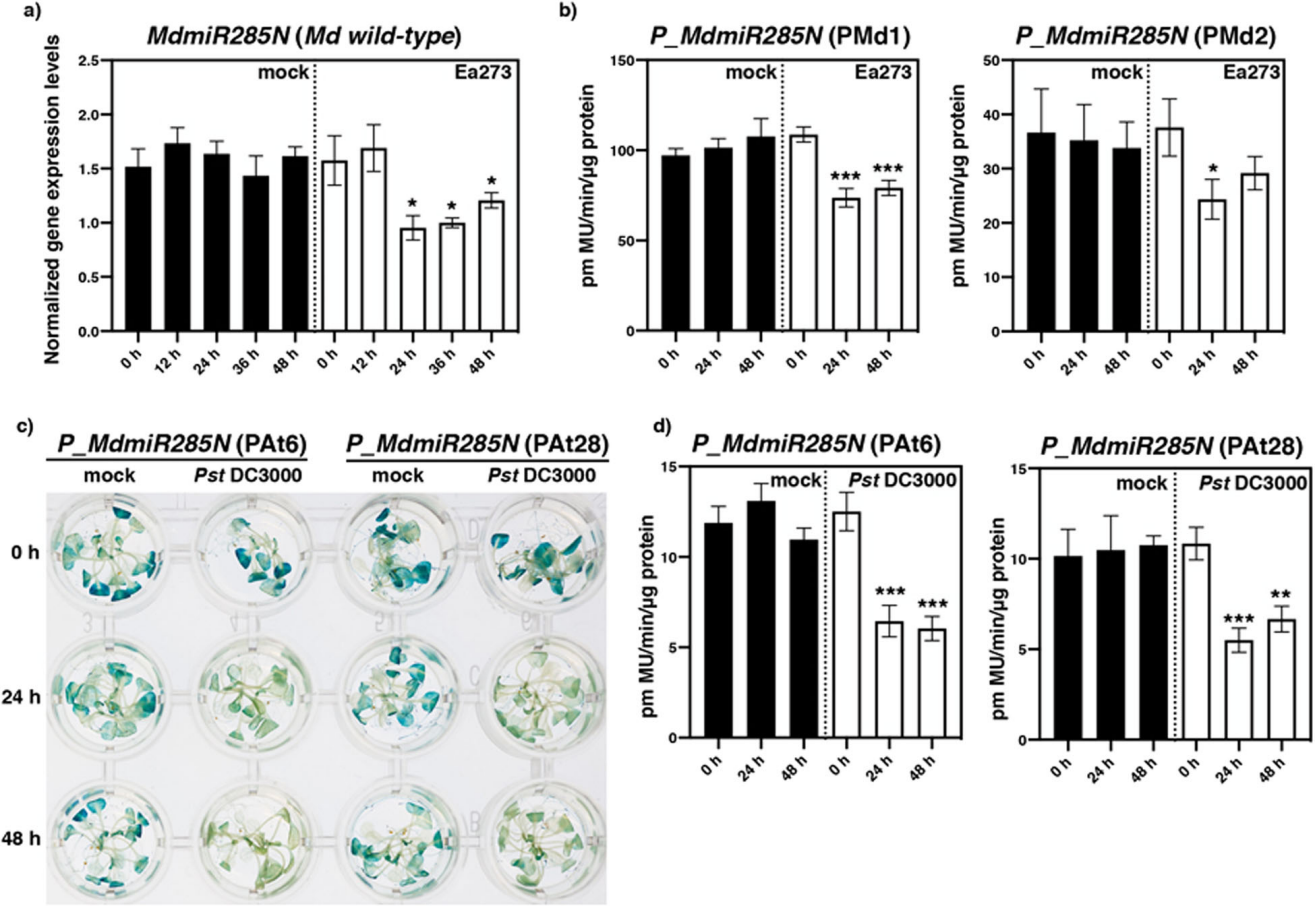
Expression pattern of *MdmiR285N* gene after host-pathogen infection in apple and *Arabidopsis thaliana* plants. **a** Transcripts levels of mature *MdmiR285N* quantified by real-time PCR in *M. x domestica wild-type* (cv. ‘Gala’) at different time points (0, 12, 24, 36, and 48 h) after treatment (mock and *E. amylovora* strain Ea273). **b** Fluorometric *MdmiR285N* Promoter-driven GUS activity in transgenic apple lines (PMd) at different time points (0, 24, and 48 h) after treatment (mock and Ea273). **c**, **d** Histochemical and fluorometric *MdmiR285N* Promoter-driven GUS activity in transgenic *A. thaliana* lines (PAt) at different time points (0, 24, and 48 h) after treatment (mock and *Pst* DC3000). Experiments were performed ex vitro (**a**) or in vitro (**b**–**d**), in duplicate (**a**, **b**, **d**) or triplicate (**c**). For each experiment, 4 (**a**) and 3 (**b**–**d**) plant biological replicates/treatment/time point were used. In (**a**) and (**b**–d), 1 biological replicate was made by pooling 3 leaf strips and 3 plantlets, respectively. Apple and *A. thaliana* wild-type plants used as negative control did not show appreciable histochemical or fluorometric GUS activity (data not shown). In graphs (**a**), (**b**), (**d**), bars indicate the mean values ± SE. Considering mock- and pathogen-treatments separately, asterisks indicate statistically significant differences of datasets from the corresponding dataset at time zero (0 h), according to one-way ANOVA followed by post hoc Dunnett’s test (*α* = 0.05)

In apple, when soil-acclimated wild-type plants used as control were mock-inoculated by leaf wounding, no significant fluctuation of mature *MdmiR285N* transcripts was detected by real-time PCR 12, 24, 36, and 48 h after the lesion (Fig. 5a). Differently, if plants experienced the bacterium, the abundance of mature *MdmiR285N* transcripts decreased significantly and specifically 24, 36, and 48 h after the application of the stress (Fig. 5a). Consistent results were obtained when the stimulatory effect of *E. amylovora* on *MdmiR285N* expression was investigated in the transgenic apple lines PMd1 and PMd2 (Fig. 5b). A decrease of GUS activity was confirmed 24 and 48 h after infection. In *A. thaliana*, a similar pattern of expression of the *MdmiR285N* promoter was observed in the transgenic lines PAt6 and PAt28 throughout *Pst* DC3000 infection (Fig. 5c, d).

## Discussion

In this study, we carried out the first functional characterization of the novel apple miRNA *MdmiR285N*, which was predicted to target 35 RNA transcripts coding for resistance proteins TIR-NBS-LRR, SNC1, and CDPK^29^. Many of these proteins accumulate within the cell after pathogen attacks and are pivotal for the activation of defense responses, while their decrease attenuates the activation of downstream defense signaling^42–45^. The presence of a complex MdmiR285N-resistance gene regulatory module able to control the plant immune system was thus hypothesized. Here, as initial characterization of *MdmiR285N* in *M. x domestica* and in the heterologous plant species *A. thaliana*, we investigated its promoter region in silico (Fig. 1, Supplementary Table S3), by histological assays (Figs. 2–4) and functional gene expression analysis in response to the bacterial pathogens *E. amylovora* and *Pst* DC3000 (Fig. 5).

Information regarding the presence of putative transcription factor (TF) binding sites (TFBSs) in a given gene promoter and their corresponding TFs is valuable for understanding potential gene regulation and biological functions. Over the past few years, different computational approaches have been developed to identify and feature DNA sequences regulating the transcription of genes^46,47^. In our work, the promoter region of *MdmiR285N* was scanned by the Plant Promoter Analysis Navigator PlantPAN 2.0^34^, using both *M. x domestica* and *A. thaliana* TFBSs databases as reference. In both plant species examined, a considerable series of putative TFBSs and corresponding TFs was identified regulating the *MdmiR285N* gene promoter during different stages of plant growth and development, and in response to multiple phytohormones signaling pathways and environmental stresses (Fig. 1, Supplementary Table S3). Taking into account that *MdmiR285N* is an endogenous miRNA of apple, our results show that its putative TFBS-based gene regulatory profile is conserved in the heterologous system *A. thaliana*, thus suggesting also that the regulation of genes involved in defense responses may be similar between the two plant species examined. This is in line with previous comparative studies showing the conservation of the regulatory networks in the promoter of MIR168 in *M. domestica* and *A. thaliana*^48^. Moreover, such a heterogeneous network of gene regulatory elements indicates that the posttranscriptional activity of MdmiR285N on its target resistance transcripts is differentially regulated during various phases of the plant life cycle and thus it is not only limited to the molecular mechanisms triggered by plant–pathogen interactions.

To validate by experimental evidences whether *MdmiR285N* expression was regulated during the plant development, GUS histochemical observations were conducted in *Prom_MdmiR285N::GFP-GUS* transgenic apple and *A. thaliana* plants and the obtained results correlated with the *in silico* data. At first, GUS analysis conducted in germinating *A. thaliana* seedlings (Fig. 3b–f) revealed that *MdmiR285N* is expressed only in roots, thus suggesting that *MdmiR285N* has function limited to root formation or nutrients uptake during the early phase of vegetative development. Differently, the expression of *MdmiR285N* drastically increases in multiple organs of *A. thaliana* during the juvenile-to-adult vegetative phase change (Fig. 3g, h). To date, it is well-documented that miRNAs play an important role in regulating vegetative phase change in plants^49,50^. In *A. thaliana* (Fig. 3g), two weeks after plant germination such a precise increase in the expression of *MdmiR285N* suggests that this miRNA may be key during the transition phase of meristems. Subsequent GUS analysis showed that *MdmiR285N* is expressed in almost all tissues of fully developed apple and *A. thaliana* plantlets (Figs. 2, 3h–l), especially in newly and growing tissues of both root and shoot systems, suggesting important roles of *MdmiR285N* in the molecular mechanisms underlying actively dividing tissues. Finally, strong expression of *MdmiR285N* was also observed in *A. thaliana* reproductive tissues and in floral organ abscission zones (Fig. 4). The role of miRNAs in the control of flowering time, floral organ identity and abscission is now reported^51,52^. Based on this information, *MdmiR285N* may be likely involved also in cellular processes responsible for plant reproduction.

In general, a close-up examination of histochemical results may propose that the vascular system is the main source of MdmiR285N. Within this context, miRNAs localized in the vascular system have often roles in plant long-distance signaling. Different studies reported the presence of specific miRNAs moving over long distances in grafts^53,54^. Moreover, miRNA movement from shoots to roots could be correlated with long‐distance signaling during nutrient starvation responses in *A. thaliana*^53,54^ or the regulation of specific developmental events in potato^55,56^. It is therefore possible that MdmiR285N acquired a long-distance signaling role. However, it is also known that many bacterial pathogens are specialized parasites of plant vascular systems^57–59^. Based on these observations, and given that MdmiR285N posttranscriptionally regulate several disease resistance proteins, its localization in the vascular system could also reflect the presence of a putative defense mechanism mediated by MdmiR285N against plant vascular pathogens.

To confirm the putative role of *MdmiR285N* in response to host-pathogen infection, *MdmiR285N* expression in wild-type apple plants and GUS activity in transgenic apple and *A. thaliana* plants were analyzed in response to *E. amylovora* (for apple) and *Pst* DC3000 (for *A. thaliana*) (Fig. 5). Overall, *MdmiR285N* appeared downregulated in both plant species examined thus suggesting an increase of its targeted disease resistance transcripts during pathogen infection. To date, many studies reported that plants are able to induce expression of disease resistance genes by suppression of the miRNA-mediated gene silencing pathway upon pathogen attack^60–64^. Within this context, a fine regulation of disease resistance proteins is also mandatory for a correct plant growth and development. Disease resistance proteins were indeed shown to have a cost to plants^65^ because if unregulated they can trigger autoimmunity in the absence of pathogen infection and inhibit plant growth^60^. Plants have thus evolved miRNA-disease resistance proteins regulatory loops as counter-mechanisms to minimize the cost of overexpression of disease resistance genes in the absence of a pathogen, and to ensure rapid induction of disease resistance proteins during pathogen invasion. This information supports our findings, suggesting a similar mechanism of action for MdmiR285N on its putative resistance transcripts, and that *MdmiR285N* may act as positive regulator of plant defense response upon plant–pathogen interactions. These observations could also explain the tissue and organ-specific expression patterns of *MdmiR285N*, according to which this miRNA was shown to be strongly induced in juvenile or developing plant tissues. The activity of MdmiR285N in those tissues is thus probably required to suppress basal defense mechanisms and allow growth and development of actively dividing tissues.

In conclusion, in this study we provide in silico and histological information regarding how *MdmiR285N* is regulated during the growth and development of *M. x domestica* and the heterologous plant species *A. thaliana*. Moreover, we demonstrate that *MdmiR285N* is downregulated in response to plant–pathogen interactions. This study sheds new light into the transcriptional regulation of *MdmiR285N* in apple, however, deeper analysis must be performed for a better understanding of its functions and to facilitate the designing of putative *MdmiR285N*-based strategies in a view of genetic engineering of apple.

## Materials and methods

### Plant materials and growth conditions

All experiments were performed with apple (*M. x domestica*) cultivar ‘Gala’ plants and *A. thaliana* ecotype ‘Columbia-0’ plants grown in a growth chamber at 24 ± 1 °C with a 16/8-h light/dark period.

In apple, in vitro propagation, in vitro roots stimulation and acclimation to soil were performed as described by Pessina et al.^66^. Before in vitro experiments (pathogen inoculation followed by Bradford and fluorometric assays, Fig. 5b; and histochemical GUS analysis, Fig. 2), to reduce putative effects of medium ingredients on the regulation of *MdmiR285N* gene and to minimize any difference with *A. thaliana* culturing, in vitro rooted plants were transferred to a Murashige and Skoog basal medium (MS) supplemented with 0.5% (w/v) sucrose and acclimated for 5 days. For ex vitro experiments (pathogen inoculation followed by gene expression analysis, Fig. 5a), soil-acclimated plants were grown at growth chamber conditions to the stage of interest.

Regarding *A. thaliana*, the maintenance of plants lines was ensured by sowing seeds in a 3:1 soil:perlite mixture and growing plants to mature stage for seed harvesting. Before in vitro experiments (see above, Figs. 3, 5c, d), harvested seeds were sterilized using 70% (v/v) ethanol x 10 min followed by 100% (v/v) ethanol x 2 min, suspended in 0.1% (w/v) agar, and vernalized for 3 days at 4 °C in the dark. Thus, seedlings were germinated and grown to the stage of interest in liquid ½MS basal medium supplemented with 0.5% (w/v) sucrose, using 24-well plates. For ex vivo histochemical GUS analysis of the inflorescence (Fig. 4), seeds were germinated in a soil:perlite mixture as previously mentioned and plants were grown to the stage of interest.

### Construction of the transformation vector

To produce the binary vector used for apple and *A. thaliana* transformations (Supplementary Tables S1, S2), genomic DNA was extracted from apple leaf tissue using the Illustra^TM^ Nucleon DNA Extraction Kit PHYTOPURE^TM^ (GE Healthcare). Extracted DNA was quantified on the NanoDrop 8000 Spectrophotometer (Thermo Fisher Scientific) and then used in a PCR aimed at amplifying 2 kb of intergenic genomic DNA sequence upstream of the transcription start site of *MdmiR285N* gene. PCR was performed on 40 ng of starting DNA using the thermocycle-3000 (Biometra), the Phusion® High-Fidelity DNA Polymerase (Thermo Fisher Scientific) and the pair of primers attB-MdmiR285N_Prom reported in Supplementary Table S4. The PCR product was directly cloned into a pENTR/D TOPO vector (Invitrogen), and subsequently the *MdmiR285N* promoter region was recombined by LR reaction (Invitrogen) into the GATEWAY^TM^ binary vector pKGWFS7^67^ in-frame with the downstream GFP-GUS gene fusion system.

### Plant transformation and identification of transgenic lines

For the production of *Prom_MdmiR285N::GFP-GUS* transgenic apple and *A. thaliana* plants, *A. tumefaciens* strain GV3101-pMP90RK^68^ competent cells were transformed by electroporation with the previously generated pKGWFS7 binary vector.

In apple (Supplementary Table S1), in vitro-propagated wild-type plantlets were transformed as described by Joshi et al.^69^, using 770 leaf explants for infections. After transformation, regenerated plants were screened for T-DNA. Genomic DNA was extracted from leaves and quantified, as previously mentioned. Thus, genomic DNA was amplified by PCR using the GoTaq® Green Master Mix 2X (Promega, Fitchburg, MA) and the pairs of primers NptII (used to detect T-DNA), MdUBQ (used as endogenous control for genomic DNA amplification), and VirG (used to verify the presence of residual *A. tumefaciens*) listed in Supplementary Table S4. The identified transgenic plants were collected and propagated in vitro.

In *A. thaliana* (Supplementary Table S2), soil-grown wild-type plantlets were transformed by the *A. tumefaciens*-mediated floral dip transformation method^70^. Following transformation, T1-independent transgenic lines were retrieved using ½MS medium supplemented with 0.5% (w/v) sucrose and 50 ng/μL kanamycin. Thus, the germination profile of the T2 offspring of the identified transgenic lines was screened on selective medium and only those lines that showed a germination ratio Kanamycin^Resistant^:Kanamycin^Susceptible^ significantly near to 3:1 were collected. Candidate lines selected in this study were grown to the T4 generation and the obtained T4 seeds were used for the experiments.

### Quantification of *NptII* copy number by Taqman real-time PCR

In apple, the investigation of the *NptII* CN (Supplementary Table S1) was performed to quantify the number of T-DNA insertion events in in vitro transgenic plants obtained by *A. tumefaciens*-mediated transformation. The experimental procedure was conducted according to the TaqMan real-time PCR method described by Dalla Costa et al.^71^. Primers and probes used for the amplification of *MdTOPO6* (endogenous gene) and *NptII* (marker gene) are listed in Supplementary Table S4.

### In silico analysis of *MdmiR285N* gene promoter sequence

To detect putative TFBSs and corresponding TFs involved in the regulation of *MdmiR285N* (Fig. 1, Supplementary Table S3), its promoter sequence (~1 kb upstream of the translation start site) was scanned by the ‘Promoter Analysis’ tool of PlantPAN 2.0 (http://PlantPAN2.itps.ncku.edu.tw; ref. ^34^), using both ‘*M. x domestica*’ and ‘*A. thaliana*’ databases as reference. The similarity score for TFBSs calling was set to 0.95. Results were downloaded and manually checked to remove putative inconsistencies. For each TFs detected, corresponding biological functions based on gene ontology information were retrieved by using both PlantPAN 2.0 and PlantTFDB 5.0 (planttfdb.cbi.pku.edu.cn) databases.

### Histochemical GUS assay

The histochemical GUS staining of apple and *A. thaliana* samples (Figs. 2–4, 5c) were carried out following the procedure described by Jefferson et al.^72^ with some variations. Samples were immersed in 90% (v/v) acetone, kept at −20 °C for 30 min, then transferred into a GUS staining solution containing 1 mM X-Gluc, 2.5 mM K_3_Fe(CN)_6_, 2.5 mM K_4_Fe(CN)_6_, 0.2% (v/v) Triton X-100 (Sigma-Aldrich), and 50 mM sodium phosphate buffer (pH 7.0). Thus, samples were vacuum infiltrated (2 and 1 min for apple and *A. thaliana*, respectively) and incubated for 12 h at 37 °C. After staining, the GUS reaction was stopped by immersing samples in a 3:1 ethanol:acetic acid solution for 6 h. Finally, samples were washed two times with 100% (v/v) ethanol for 12 h to remove the chlorophyll, and subsequently conserved in 70% (v/v) ethanol. Imaging of stained tissues was performed using a full-frame DLSR camera with a 100 mm macro lens (Nikon), a Axio Imager 2 microscope (ZEISS), and a MZ16 F stereomicroscope (LEICA).

### Pathogen inoculation

For ex vivo inoculations of apple (Fig. 5a), wild-type plantlets (grown for 3 weeks after acclimation to soil) were inoculated according to the scissor inoculation method described by Desnoues et al.^73^, using *E. amylovora* strain Ea273. *E. amylovora* was grown at 28 °C x 24 h in liquid KADO medium^74^ supplemented with 0.3 g/L MgSO_4_. Following growth, the bacterial cell density was measured with a BioPhotometer (Eppendorf, Hamburg, Germany), thus the inoculum solution was prepared by adjusting bacterial concentration to 1 × 10^9^ CFU/mL with 0.05 M potassium phosphate buffer (pH 6.5). The three youngest leaves of plants actively growing were transversally cut using scissors dipped in the bacteria suspension or potassium phosphate buffer (mock) as mechanical damage control. After treatment, plants were maintained at growth chamber conditions and subsequently sampled. Approximately 5-mm-wide leaf strips, parallel to the inoculation cut, were collected at 0, 12, 24, 36, and 48 h postinoculation. Samples were frozen in liquid nitrogen and kept at −80 °C for the further RNA extraction.

For in vitro inoculations of apple and *A. thaliana* (Fig. 5b–d), the procedure was carried out according to the flood-inoculation technique described by Ishiga et al.^75^ with some modifications. For *A. thaliana*, inoculations were performed using *Pst* DC3000^76^. The bacterial pathogen was grown at 28 °C on Luria–Bertani (LB) medium x 24 h. After growth, bacterial was suspended in sterile distilled H_2_O and the bacterial cell density (OD_600_) was measured as previously mentioned. Thus, bacterial inoculation solution (1 × 10^6^ CFU/mL), prepared in sterile distilled H_2_O containing 0.005% Silwet L-77 (Sigma-Aldrich), was poured into 24-well plates containing 3-week-old *A. thaliana* seedlings. Plants used as control were treated using a mock solution prepared according to the previous inoculation solution without the bacteria. After 3 min of immersion and low agitation at 50 rpm, inoculation solutions were discarded and the liquid culture medium was replaced. Treated plants were maintained at growth chamber conditions and sampling was performed at 0, 24, and 48 h postinoculation. Collected plants were directly used in the histochemical GUS procedure as previously described, or frozen in liquid nitrogen and conserved at −80 °C for the further Bradford and fluorometric MUG assays. For apple, *E. amylovora* was grown as previously described and the inoculum solution was prepared by adjusting bacterial concentration to 1 × 10^6^ CFU/mL with 0.05 M potassium phosphate buffer (pH 6.5) and 0.005% Silwet L-77. For control experiments, a mock solution was made as the inoculum solution without the bacteria. The treatment was performed by pouring inoculation or mock solutions into baby jars containing 3-week-old apple plantlets. After 6 min of flood-treatment with low agitation at 50 rpm, solutions were discarded and corresponding treated plants were kept at growth chamber conditions. As for *A. thaliana*, plants were sampled at 0, 24, and 48 h postinoculation and conserved at −80 °C for the subsequent Bradford and fluorometric MUG assays.

### Real-time PCR

For the expression analysis of mature *MdmiR285N* transcripts (Fig. 5a), the experimental procedure was conducted according to the protocol of Varkonyi-Gasic et al.^77^ with minor variations. Samples were ground with a mortar and pestle chilled with liquid nitrogen, and the resulting powder was used for total RNA extraction using the Spectrum^TM^ Plant Total RNA Kit (Sigma-Aldrich). Extracted RNA was quantified on the NanoDrop 8000 Spectrophotometer (Thermo Fisher Scientific) and subsequently treated with DNase I (Sigma-Aldrich) to remove genomic DNA contamination. One microgram of DNase-treated RNA was applied for the *MdmiR285N*-specific cDNA synthesis using the Superscript III RT kit (Invitrogen), the MdmiR285N-stemloop primer (Supplementary Table S4), and a pulsed reverse transcription (1 cycle of 16 °C for 30 min; 60 cycles of 30 °C for 30 s, 42 °C for 30 s, and 50 °C for 1 s; and 1 cycle of 70 °C for 15 min). In parallel, *MdU6* and *MdACT2* genes (used as internal controls) were retrotranscribed according to the manufacturer’s instructions of the Superscript III RT kit (Invitrogen). After RT reaction, the produced cDNA was diluted ten times and then used in real-time PCR reactions conducted in a 96-well plate with 5 ng of starting cDNA, the SsoAdvanced^TM^ Universal SYBR® Green Supermix (Bio-Rad) and the couples of primers MdU6, MdACT2, and MdmiR285N (F, R) reported in Supplementary Table S4. Real-time PCRs were performed on a C1000 thermal cycler (Bio-Rad) equipped with CFX96 real-time PCR detection system (Bio-Rad) and a data analysis software CFX Maestro (Bio-Rad).

### Bradford and fluorometric MUG assays

Collected apple and *A. thaliana* in vitro plantlets were ground with a mortar and pestle chilled with liquid nitrogen. Hundred milligrams of the resulting powder was used for the quantification of GUS activity (Fig. 5b, d) according to the experimental procedure described by Dalla Costa et al.^78^.

### Statistical analysis

Regarding the statistical analysis of segregation T-DNA loci data in *A. thaliana* (Supplementary Table S2), the Chi-square (*X*^2^) test was used to assess the differences between the observed values and the expected values.

For gene expression and GUS activity quantitative data (Fig. 5), the statistical analysis was conducted with the Dell^TM^ Statistica^TM^ Software version 13.1, considering datasets of mock- and pathogen-treatments separately. A one-way ANOVA followed by post hoc Dunnett’s test was used to assess differences between datasets and the corresponding control dataset (0 h). Statistics was performed with *α* = 0.05.

## Supplementary information

Supplementary information
