# Supplementary material for: Transcriptional regulation of MdmiR285N microRNA in apple (Malus x domestica) and the heterologous plant system Arabidopsis thaliana
Source: Hortic Res. 2020 Jul 1;7:99. doi: 10.1038/s41438-020-0321-5 (PMC7326934; doi:10.1038/s41438-020-0321-5)
Supplement: Supplementary file 1 — Supplementary information [file 41438_2020_321_MOESM1_ESM.docx]

**Table S1: Summary of transformation results and selection of transgenic lines in apple (*Malus x domestica*).** The transformation efficiency was calculated by dividing the number of regenerated plants positive for *NptII* and negative for *VirG* by the number of leaf explants infected. *MdUBQ* gene was amplified to test the integrity of genomic DNA used in PCRs. The *NptII* copy number (CN) of each selected plant line is the mean ± SD of 4 biological replicates. *Neomycin phosphotransfare II* gene **(***NptII*); *Ubiquitin* gene (*MdUBQ*); *A. tumefaciens Virulence G* gene (*VirG*).

| Apple cultivar | Transformation  vector | No. of leaf explants infected | No. of regenerants collected | PCR screening | | | | Transformation efficiency | Selected lines: Line ID (*NptII* CN) |
| --- | --- | --- | --- | --- | --- | --- | --- | --- | --- |
|  |  |  |  | **No. of regenerants tested** | **No. of positive regenerants** | | |  |  |
|  |  |  |  |  | ***NptII*** | ***MdUBQ*** | ***VirG*** |  |  |
| Gala | pKGWFS7 | 770 | 6 | 5 | 5 | 5 | 1 | 0.5 | PMd1 (2.01±0.12)  PMd2 (1.00±0.45) |

**Table S2: Summary of transformation results and selection of transgenic lines in *Arabidopsis thaliana*.** Chi-square (*X*^2^) test was used to determine the probability (*P*) of which the deviation of the observed value (ratio tested) from the expected value (ratio R:S=3:1) was due to chance. Kanamycin (Kan); Resistant (R); Susceptible (S).

| *Arabidopsis thaliana* ecotype | Transformation  vector | Selection of transformed plants with single T-DNA insertion event | | | | | *X ^2^* | Signification level (*P*) |
| --- | --- | --- | --- | --- | --- | --- | --- | --- |
|  |  | **No. of analyzed**  **T1 lines** | | **Screening of T2 generation** | | |  |  |
|  |  |  | **Line ID** | **Kan^R^**  **(No. seeds)** | **Kan^S^**  **(No. seeds)** | **Ratio tested**  **(R:S=3:1)** |  |  |
| Col-0 | pKGWFS7 | 40 | PAt2  PAt6  PAt13  PAt26  PAt27  PAt28  PAt32  PAt36 | 135  112  140  186  106  140  153  146 | 54  46  61  48  44  39  47  63 | 2.5:1  2.4:1  2.3:1  3.9:1  2.4:1  3.6:1  3.3:1  2.3:1 | 1.286  1.426  3.066  2.513  1.502  0.985  0.240  2.949 | 0.25  0.23  0.08  0.11  0.22  0.32  0.62  0.08 |

**Table S3: List of TFBSs identified in the promoter region of *MdmiR285N* gene.** TFBSs calling was performed in 1 kb of *MdmiR285N* promoter sequence upstream of the transcription start site (ATG), using separately the *Malus x domestica* and *Arabidopsis thaliana* databases of PlantPAN 2.0 (http://PlantPAN2.itps.ncku.edu.tw). A sequence similarity score higher than 0.95 was used. Information regarding TFs families, TFs and the associated biological process were retrieved by comparing the results obtained from both PlantPAN 2.0 and PlantTFDB 5.0 (planttfdb.cbi.pku.edu.cn) databases.

| ***MALUS x DOMESTICA* DATABASE** | | | |
| --- | --- | --- | --- |
| **TFBS sequence** | **Position (0..1052-ATG)** | **TFs Family** | **TF gene ID (Biological Process)** |
| gAGATTcgag | 724 | ARR-B | MDP0000124301 (response to cytokinins); MDP0000290818 (response to cytokinins and water deprivation; regulation of root meristem growth, chlorophyll biosynthetic process and anthocyanin metabolic process; development of shoot, primary root and seed) |
| ccCACGTgt | 778 | bHLH | MDP0000029168 (response to dessication, wounding, abscisic acid, jasmonate signaling pathway and chitin; regulation of flavonoid biosynthetic process); MDP0000138721 (phytochrome signaling pathways); MDP0000198404 (photrotransduction; ethylene biosynthetic process; response to light stimulus; regulation of auxin biosynthetic and signaling pathways) |
| ccCACGTgtg | 778 | bZIP | MDP0000144105 and MDP0000177486 (response to cold, water deprivation, salt stress, abscisic acid, gibberellin, chitin; seed germination and development); MDP0000286846 (response to light stimulus) |
| tAGTGTt | 101 | C2H2 | MDP0000762756 (response to oxidative stress, cold, water deprivation, wounding, high light intensity, salt stress, abscisic acid, chitin; photoprotection) |
| tACACTa | 244 |  |  |
| aTAACTc | 88 |  | MDP0000225209 (response to hormone-mediated signaling pathways; specification of floral organ identity; regulation of cell proliferation) |
| gtTTAAAa | 177 | CPP | MDP0000182903 and MDP0000233007 (regulation of development of both male and female reproductive tissues) |
| aATAAAag | 509 | CSD | MDP0000155077 and MDP0000202739 (response to cold-shock) |
| tTATTTtg | 544 |  |  |
| aATAAAaa | 561 |  |  |
| tagTGCATca | 912 | EIL | MDP0000144922 (involved in the ethylene response pathway) |
| ggaGATTCgag | 723 | G2-like protein | MDP0000202657 (phosphate signaling in roots); MDP0000229587 (regulation of vascular development; phloem and xylem differentiation) |
| gAGATCaaag | 469 | GATA | MDP0000137305 (circadian rhythm and seed germination); MDP0000220844 (response to light stimulus) |
| ttcATTAAtt | 327 | HD-ZIP | MDP0000046392 (seed gemination; epidermal cell differentiation; maintenance of floral organ identity; cotyledon development); MDP0000125182 and MDP0000125184 (response to drought stress; regulation of jasmonate and auxin biosynthesis and signaling; lateral root formation) |
| gacATTAAaa | 483 |  |  |
| aaacAAAAAgaaaaataaaaa | 548 | MIKC_MADS | MDP0000013331 (meristem structural organization; maintenance of floral meristem identity and determinancy) |
| agTAGGTa | 1025 | MYB | MDP0000135594 (response to ethylene, abscisic acid, auxin and biotrophic phatogens); MDP0000175918 (response to wounding, salt stress, abscisic acid and salicylic acid; seed trichome elongation) |
| catTATCCc | 448 | MYB-related | MDP0000140324 and MDP0000230601 (regulation of drought-responsive genes; stomatal closure; response to abscisic acid); MDP0000758053 (response to salt stress, ethylene, auxin, abscisic acid, gibberellin, salicylic acid and jasmonate; regulation of anthocyanin biosynthetic process) |
| aaGTCAAtc | 253 | NAC | MDP0000120881 (anther and pollen development; seed development and morphology); MDP0000132623 (jasmonate-mediated response to biotrophic pahtogens); MDP0000180683 (regulation of ethylene biosynthesis; seed development and morphology); MDP0000481448 (fruit ripening; flower development; leaf senescence); MDP0000802924 (stress-induced signaling); MDP0000868419 (response to jasmonate; seed morphogenesis and embryonic development) |
| acTTGACtg | 525 |  |  |
| acttGCACCa | 766 | TCP | MDP0000120671 (leaf morphogenesis and cell differentiation); MDP0000130524 (ovule development; control of shoot organs morphogenesis; response to light stimulus) |
| ttTAACCa | 386 | Trihelix | MDP0000257669 (response to light signals) |
| aaaGTCAAtc | 252 | WRKY | MDP0000119590 (regulation of elicitor-responsive genes); MDP0000121669 (jasmonic acid mediated signaling pathway; defense response to fungus); MDP0000128464 (defense reponse to necrotrophic and biotrophic pathogens); MDP0000130400 (regulation of jasmonic acid signaling pathway; regulation of systemic resistance) |
| cTTGACtg | 526 |  |  |
| ***ARABIDOPSIS THALIANA* DATABASE** | | | |
|  |  |  |  |
|  |  |  |  |
| **TFBS Sequence** | **Position (0..1052-ATG)** | **TF Family** | **TF gene ID (Biological process)** |
| acGGATCcac | 758 | ARR-B | AT1G67710 (response to cytokinin; cytokinin-activated signalin pathway; regulation of root growth); AT2G01760 (cytokinin-activated signaling pathway) |
| ctgcAATCTc | 867 |  | AT4G18020 (regulation of circadian rhythm) |
| gaGATTCgag | 724 |  | AT3G16857 (response to water deprivation; regulation of root growth, chlorophyll biosynthetic process, anthocyanin metabolic process, cytokinin-activated signaling pathway and seed growth; shoot system development; primary root development); AT2G01760 (cytokinin-activated signaling pathway) |
| ttcccACGTGtgaa | 776 | BES1 | AT1G19350 (brassinosteroid mediated signaling pathway; defense response to bacterium); AT1G75080 (brassinosteroid mediated signaling pathway; regulation of growth; seed development; plant ovule development) |
| cccACGTGt | 778 | bHLH | AT1G10120 (response to light; regulation of growth); AT1G18400 (positive regulation of shade avoidance); AT1G25330 (brassinesteroid mediated pathway); AT1G26260 (response to light; positive regulation of flower development; regulation of growth); AT1G59640 (petal morphogenesis); AT1G68920 (regulation of growth); AT1G73830 (response to brassinosteroid, auxin, ethylene and abscisic acid); AT3G23690 (response to light; positive regulation of flower development); AT5G50915 (response to gibberellin); AT5G08130 (brassinosteroid mediated signaling; positive regulation of shade avoidance); AT1G09530 (phototransduction; de-etiolation; gibberellic acid mediated signaling pathway); AT2G46970 (shade avoidance; light signaling pathway) |
| tccACTTGc | 763 |  | AT1G05805 (regulation of stomatal movement); AT1G35460 (cuticle development); AT1G51140 (regulation of stomatal movement; cuticle development; regulation of flowering); AT2G42280 (regulation of flowering); AT2G43140 (response to abscisic acid stimulus) |
| cccACGTGtg | 778 | bZIP | AT1G49720 (response to abscisic acid); AT3G19290 (response to water deprivation and salt stress; abscisic acid-activated signaling pathway; regulation of leaf senescence and chlorophyll catabolic process); AT1G45249 (response to water deprivation and salt stress; abscisic acid-activated signaling pathway); AT2G46270 (response to abscisic acid); AT4G36730 (regulation of hydrogen peroxide metabolic process; regulation of cell aging); AT4G01120 (response to light); AT2G36270 (response to cold, water deprivation, salt stress, abscisic acid and gibberellin; pollen and seed maturation; response to chitin); AT3G54620 (seed maturation) |
| agCAGCTgcc | 798 | C2H2 | AT2G17180 (pollen sperm cell differentiation) |
| aTAACTc | 88 |  | AT5G06070 (response to cold; regulation of meristem structural organization; petal development) |
| tACACTa | 244 |  | AT1G27730 (response to oxidative stress, cold, water deprivation, wounding, high light intensity, salt stress and chitin; abscisic acid mediated signaling); AT5G04340 (response to chitin; regulation of root development); AT5G43170 (response to chitin, cold and salt stress); AT3G19580 (response to water deprivation, salt stress and chitin; abscisic acid mediated signaling); AT5G04340 (response to chitin; root development); AT5G43170 (response to chitin, cold and salt stress) |
| tAGTGTt | 101 |  |  |
| gcaAATTTcaaag | 74 | CPP | AT4G14770 (development of both male and female reproductive tissues) |
| gtTTAAAa | 177 |  |  |
| ttcttAAATTtac | 410 |  |  |
| aATAAAaa | 509 | CSD | AT2G21060 (embryo and fruit development; response to cold stress); AT4G38680 (response to cold and dessication; abscisic acid mediated signaling; seed development; vegetative to reproductive phase transition of meristem) |
| aATAAAaa | 561 |  |  |
| aATAAAac | 544 |  |  |
| aaAAAAGgac | 125 | Dof | AT5G39660 (flower development) |
| aaaTCTTTtt | 379 |  | AT5G65590 (guard cell differentiation; stomatal movement; regulation of cell wall pectin metabolic process) |
| aCTTTAacg | 192 |  | AT4G38000 (floral organ abscission) |
| aCTTTAacg | 221 |  |  |
| aCTTTTaac | 818 |  |  |
| taAAAAGtca | 249 |  | AT2G46590 (response to cold; response to light stimulus; seed germination); AT5G39660 (flower development); AT4G38000 (floral organ abscission); AT5G62940 (procambium histogenesis; phloem or xylem histogenesis); AT3G47500 (flower development) |
| taAAAGAgat | 464 |  | AT5G65590 (guard cell differentiation; stomatal movement; regulation of cell wall pectin metabolic process) |
| tagTGCATca | 912 | EIL | AT3G20770 (response to hypoxia and iron ion; ethylene-activated signaling pathway; defense response to bacterium); AT5G21120 and AT5G65100 (response to iron ion; ethylene-activated signaling pathway) |
| ggaGATTCgag | 723 | G2-like protein | AT2G40970 (response to cold); AT3G46640 (regulation of circadin rhythm); AT5G59570 (regulation of circadian rhythm and flower development); AT2G03500 (response to temperature stimulus; gibberellic acid mediated signaling pathway; flower development; negative regulation of long-day photoperiodism; flowering); AT1G68670 (response to abscisic acid); AT4G28610 (regulation of circadian rhythm; response to high light intensity; response to phosphate starvation); AT1G79430 (development of flower, phloem, and xylem); AT3G24120 (cellular response to phosphate starvation) |
| gAGATCaaag | 469 | GATA | AT2G45050 and AT3G51080 (response to light stimulus); AT5G25830 (circadian rhythm; response to light stimulus) |
| gacATTAAaa | 483 | HD-ZIP | AT1G05230 and AT1G17920 (trichome morphogenesis; maintenance of floral organ identity); AT2G32370 (cotyledon development); AT3G61150 (maintenance of floral organ identity); AT4G21750 (seed germination; cotyledon development); AT5G46880 (maintenance of floral organ identity) |
| ttcATTAAtt | 327 |  |  |
| aaacAAAAAgaaaaataaaaa | 548 | MIKC-MADS | AT4G22950 (flower development; vernalization response); AT4G24540 (response to gibberellin; maintenance of floral and inflorescence meristem identity; vernalization response); AT5G51860 and AT5G51870 (regulation of flower development); AT5G60910 (positive regulation of flower development; maintenance of inflorescence meristem identity; fruit development); AT5G62165 (regulation of flower development; abscission; leaf and floral organ senescence); AT1G26310 (positive regulation of flower development; floral meristem determinancy); AT2G14210 (response to nutrient and nitrate; lateral root development); AT2G22630 (positive regulation of long-day photoperiodism; flowering); AT2G45650 (embryo sac development; positive regulation of flower development; specification of carpel identity; vegetative to reproductive phase transition of meristem; floral meristem determinancy; floral organ development; stamen formation; floral whorl structural organization; specification of floral organ number; integument development; seed growth); AT2G45660 (response to cold; response to gibberellin; positive regulation of flower development; maintenance of inflorescence meristem identity); AT3G57230 (stomatal lineage progression; long-day photoperiodism; flowering); AT3G57390 (pollen development; negative regulation of flower development; negative regulation of short-day photoperiodism; flowering); AT3G61120 (pollen and plant ovule development); AT4G09960 (seed development; plant ovule development; regulation of double fertilization forming a zygote and endosperm; seed trichome differentiation); AT4G11880 (flower development; maintenance of floral meristem identity; regulation of root meristem growth; vegetative to reproductive phase transition of meristem; regulation of auxin polar transport) |
| tccTTATCat | 292 | MYB | AT5G04760 (regulation of flower morphogenesis) |
| agTAGGTa | 1025 |  | AT2G16720 (response to salt stress and salicylic acid; regulation of flavonol biosynthetic process); AT4G09460 (response to gibberellin, salicylic acid and jasmonic acid; seed trichome elongation); AT4G34990 (response to salt stress, ethylene, abscisic acid, salicylic acid and jasmonic acid); AT4G38620 (response to salicylic acid, jasmonic acid and UV-B; secondary cell wall biogenesis involved in seed trichome differentiation) |
| caTTATCccc | 448 | MYB-related | AT1G70000 (response to salt stress, ethylene, auxin, abscisic acid, gibberellin; salicylic acid and jasmonic acid; regulation of anthocyanin biosynthetic process); AT3G16350 (response to salt stress, ethylene, auxin, gibberellin and jasmonic acid); AT5G47390 (response to salt stress, ethylene, abscisic acid, gibberellin; salicylic acid and jasmonic acid; leaf development) |
| aaCCCTAg | 899 |  | AT3G49850 and AT5G67580 (response to salt stress, ethylene, auxin, gibberellin, salicylic acid and jasmonic acid) |
| aaGTCAAtc | 253 | NAC | AT1G01720 (response to wounding; negative regulation of abscisic acid-activated signaling pathway); AT1G52880 (regulation of embryonic development; seed morphogenesis; integument development); AT1G52890 (response to water deprivation); AT1G69490 (fruit ripening; flower development; leaf senescence); AT3G15500 (jasmonic acid mediated signaling pathway; response to water deprivation); AT3G15510 (response to jasmonic acid; regulation of embryonic development; seed morphogenesis; integument development); AT4G27410 (response to water deprivation; response to abscisic acid) |
| acTTGACtg | 525 |  |  |
| acttGCACCa | 766 | TCP | AT1G30210 (leaf morphogenesis; regulation of development); AT1G67260 (flower development); AT1G68800 and AT3G18550 (regulation of secondary shoot formation); AT3G02150 (leaf morphogenesis; regulation of development; regulation of light stress response) |
| tTAACCaact | 387 | Trihelix | AT1G13450 (response to light signal) |
| aaGTCAAtcc | 253 | WRKY | AT1G18860, AT1G29280 and AT1G29860 (regulation of elicitor-induced response); AT1G55600 (regulation of elicitor-induced response, endosperm development); AT1G62300 (ethylene-activated signaling pathway; response to chitin and phosphate starviation); AT2G34830 (pollen development; embryo development ending in seed dormancy); AT2G40750 (response to salicylic acid; regulation defense response to pathogens; regulation of leaf senescence); AT2G46400 (response to chitin; lateral root development); AT3G56400 (systemic acquired resistance, salicylic acid mediated signaling; induced systemic resistance, jasmonic acid mediated signaling; response to chitin; ; regulation of leaf senescence); AT4G23810 (response to salicylic acid; defense response to bacterium, incompatible interaction; leaf senescence); AT4G39410 (regulation of lignin biosynthetic process); AT5G15130 and AT5G22570 (salicylic acid mediated signaling pathway and defense response to bacterium); AT5G26170 (jasmonic acid mediated signaling pathway; defense response to fungus); AT5G45050 (defense response to biotic stress); AT5G45260 (cell death; defense response to bacterium, incompatible interaction) |
| cTTGACtg | 526 |  |  |

| **Primers and probes** | **Sequences (5’-3’)** |
| --- | --- |
| MdUBQ | F: CATCCCCCCAGACCAGCAGA  R: ACCACGGAGACGAAGCACCAA |
| VirG | F: GCCGGGGCGAGACCATAGG  R: CGCACGCGCAAGGCAACC |
| MdTOPO6 | F: TGTGGAAGGAGATCAGCGCA  R: CGCGTTGCTTCTTTGCTGCA |
| MdTOPO6_probe | FAM-5′-ACATGCCAACAGGAACAATCACA-3′-TAMRA |
| NptII | F: CTTGCCGAATATCATGGTGGAA  R: GGTAGCCAACGCTATGTCCTGA |
| NptII_probe  attB-MdmiR285N_Prom  MdmiR285N  MdU6  MdACT2 | FAM-5′-TTCTGGATTCATCGACTGTGGC-3′-TAMRA  F: CCATCTTAGGCTCCAAAACTCGCAATCAAG  R: CTCCTACCTACTAGCTTTAGCCAAAGAG  Stemloop: GTTGGCTCTGGTGCAGGGTCCGAGGTATTCGCACCAGAGCCAACTATGGT  F: GTTTGGGTAAGTTCATCCAAAC  Universal R: GTGCAGGGTCCGAGGT  F: GATAAAATTGGAACGATACAG  R: GGACCATTTCTCGATTTATGCG  F: TGGTGAGGCTCTATTCCAAC  R: TGGCATATACTCTGGAGGCT |

**Table S4: List of primers and probes used during the analysis.** AttB-MdmiR285N_Prom primers were used to isolate the promoter sequence (2 kb) of *MdmiR285N* gene. NptII, MdUBQ and VirG primers are related to the PCR-based screening of putative transgenic plant lines. NptII and MdTOPO6 primers and probes sets were used for the quantification of T-DNA copy number by Taqman real-time PCR. MdU6, MdACT2 and MdmiR285N primers sets were used for the expression analysis of mature *MdmiR285N* by real-time PCR.
